# Senescence-Mediated Redox Imbalance in Liver and Kidney: Antioxidant Rejuvenating Potential of Green Tea Extract

**DOI:** 10.3390/ijerph19010260

**Published:** 2021-12-27

**Authors:** Yu-Wen Hsu, Wen-Kang Chen, Chia-Fang Tsai

**Affiliations:** 1 Department of Optometry, Da-Yeh University, Changhua 515006, Taiwan; yuwen@mail.dyu.edu.tw; 2 Department of Applied Cosmetology, National Tainan Junior College of Nursing, Tainan 700007, Taiwan; yayen0619@gmail.com

**Keywords:** aging, antioxidant enzyme, catechins, green tea, oxidative stress

## Abstract

This study investigates the catechin composition and protective effect of green tea extract on senescence-mediated redox imbalance in the livers and kidneys of aged mice. The results showed that the seven catechins in the green tea extract analyzed in this study could be completely separated within 30 min and the main components of catechins in green tea extract were EGCG, EGC and ECG. In terms of the anti-senescence effects of green tea extract, green tea extract supplementation at doses of 125, 625 and 1250 mg/kg for 4 weeks significantly alleviated the senescence-mediated redox imbalance, as exhibited from significantly (*p* < 0.05) reduced thiobarbituric acid-reactive substances (TBARS) and protein carbonyls levels in the serum, and increased glutathione (GSH) and total thiols contents in the plasma. Additionally, hepatic and renal protein carbonyls levels were significantly diminished (*p* < 0.05) and the activities of superoxide dismutase (SOD), catalase, glucose-6-phosphate dehydrogenase (G6PD), glutathione peroxidase (GSH-Px) and glutathione reductase (GSH-Rd) in the liver and kidney were remarkably elevated (*p* < 0.05). Overall, these results clearly show that green tea extract exhibits extremely potent protective effects against senescence-mediated redox imbalance in the livers and kidneys of mice by inhibiting oxidative damage of lipids and proteins and increasing the activities of antioxidant enzymes in organs.

## 1. Introduction

Senescence is a stage of growth for almost all organisms. It is an exceedingly complex physiological change, which is mainly characterized by the gradual degradation of the functions of various organs. One of the major hypotheses of aging is the oxidative stress theory, which shows that the increase in the production of reactive oxygen species (ROS) in organisms leads to an increase in lipid peroxidation and oxidation-related reactions, and ultimately results in oxidative damage [1]. ROS participate not only in the progression of normal aging but also play a role in many age-related degeneration processes. Fortunately, the organism has developed a variety of defense systems to control ROS levels and to limit the generation of oxidative injury. A principal defense strategy for preventing and mitigating aging-related oxidative damage is to inhibit the generation of reactive metabolites and diminish lipid peroxidation by raising the levels of the effectual constituent’s endogenous antioxidant defense system, such as glutathione (GSH), superoxide dismutase (SOD) and catalase [2,3]. Supplementing natural antioxidants in the daily diet has been confirmed to effectively assist the endogenous antioxidant defense system in fighting the oxidative stress that occurs during the aging process. Their role is to reduce reactivity through specific interactions or synergistic effect with reactive metabolites, and to further prevent or slow down the aging phenomenon caused by oxidative stress on various organs [4].

Green tea (*Camellia sinensis* (L), O.Kuntze, Theaceae) is one of the most fashionable drinks in the world, especially in Asian society [5]. A subacute toxicity study report of green tea extract stated that drinking green tea every day is safe and has no adverse effects in mice [6]. Green tea has been proven to have many useful biological effects that are attributed to its rich polyphenolic active substances, especially catechins and catechin derivatives, including (−) -epigallocatechin-3-gallate (EGCG), epigallocatechin (EGC) and epicatechin-3-gallate (ECG) [7].

A number of epidemiological and experimental studies showed that green tea catechins have been provided with a variety of physiological and medicinal properties and can be used not only a health-preserving beverage, but also a raw material for health foods and nutritional supplements in traditional diets. Many scientific documents have confirmed many medicinal effects of green tea catechins, such as antioxidant, antibacterial, anti-inflammatory and anti-tumor activities [7,8,9]. Furthermore, green tea extract had a protective effect on ethanol-induced oxidative stress in different tissues (including brain, liver and blood), which might be attributed to its ability to enhance the antioxidant defense system in experimental animals [10,11]. Recently, our group proved that green tea extract had a protective effect in decreasing oxidative stress, restoring the activity of antioxidant enzymes, and diminishing the incidence of hepatic fibrosis [12]. However, there are few animal models available for green tea to study age-related oxidative damage in the liver and kidneys. Hence, the purpose of this study was to explore the effectiveness of green tea, an easily available source of water-soluble antioxidants in daily diets, to diminish aging-mediated redox imbalances in the liver and kidneys of aged mice.

## 2. Materials and Methods

### 2.1. Chemicals

Catechin standards (purity ≥ 95%), including (+) -gallocatechin (GC), (−) -epigallocatechin (EGC), (+) -catechin (C), (−) -Gallocatechin gallate (EGCG), (−) -epicatechin (EC), (−) -gallocatechin gallate (GCG) and (−) -Epicatechin gallate (ECG) were purchased from Sigma Chemical Company (St. Louis, MO, USA). Analytical grade orthophosphoric acid and methanol were obtained from Merck (Darmstadt, Germany). All analytical grade reagents used were purchased from local chemical companies.

### 2.2. Materials

Green tea extract made from the leaves of *C. sinensis* was obtained from AGV Co., Ltd. (Chiayi City, Taiwan) previously described by Hsu et al. [5]. Briefly, the green tea extraction solution was prepared by adding 5 g of tea leaves to 500 mL of boiling water and soaking for 30 min. The extract solution was filtered and cooled to room temperature, and then 500 mL of boiling water was added to the previous tea leaves and soaked for 30 min, then the extraction solution filtered out and combined with the first extraction solution. The green tea extract solution was stored in a freezer at −80 °C for 24 h and then dried in a freeze dryer (−42 °C, below 133 × 10^−3^ mbar) for 48 h. After freeze-drying, the samples were crushed to form a powder and immediately stored at −20 °C for further experiments.

### 2.3. Determination of Polyphenol Contents in Green Tea Extract

Determination of polyphenol contents in green tea extract was carried out using a high-performance liquid chromatography (HPLC) system (Waters e2695, Waters Co., Milford, MA, USA) that consisted of a vacuum degasser, quaternary pump, photodiode array detector, thermostatted column compartment and a C18 reversed phase column (4.6 × 250 mm, 5-μm particle size; Gemini 5μ C18 110 Å, Phenomenex^®^) (Torrance, CA, USA), as previously described [13]. The mobile phases consisted of eluent A (deionised water containing 0.1% (*v*/*v*) orthophosphoric acid) and eluent B (methanol containing 0.1% (*v*/*v*) orthophosphoric acid). The mobile phase elution gradient at a flow rate of 1 mL/min was as follows: 0–5 min, 20% eluent B; 5–7 min, linear gradient of 20–24% eluent B; 7–10 min, 24% eluent B; 10–20 min, linear gradient of 24–40% eluent B; 20–25 min, linear gradient of 40–50% eluent B. The post-run time was 5 min. The samples were injected using a manual injection valve (10 μL injection volume, 0.5–100 ng) and were detected with a diode array detector at 280 nm. The identification and quantification of polyphenol contents in green tea extract was carried out by the comparison of retention time and absorption spectra of unknown peaks with reference standards, which were used to establish the standard calibration curves.

### 2.4. Animals

Male ICR mice, weighing approximately 22 to 28 g (young, 8 weeks old) and 38 to 46 g (aged, 52 weeks old), were purchased from the BioLASCO Taiwan Company and were isolated and adapted to their environment for seven days before experimentation. Mice were taken care of under standard research facility states of a 12 h light-dark cycle in a temperature of 23 ± 2 °C and relative humidity of 50 ± 5% controlled room. All mice had free access to food and water. Our Institutional Animal Care and Use Committee (IACUC) approved all animal experiments in this study. The animals were well cared for in accordance with institutional ethical guidelines.

### 2.5. Treatment

Group I served as the young control and was orally administered distilled water (vehicle) daily for 4 weeks. To investigate senescence-related oxidative stress in the livers and kidneys of mice, mice of Groups II, III, IV and V were composed of aged mice and the mice were randomly divided into four groups consisting of 10 mice in each group. Group II served as the aged control and was orally administered distilled water daily. Groups III, IV and V were orally administered green tea extract dissolved in distilled water at doses of 125, 625 and 1250 mg/kg, respectively, daily for 4 weeks. All animals were sacrificed at the end of experiment and blood was collected into heparinized tubes (50 U/mL) for evaluation of serum lipid peroxidation and protein carbonyls in serum and GSH and total thiol levels in plasma. For serum preparation, the blood was allowed to clot by leaving it undisturbed at room temperature for 30 min after collection of the whole blood. Then, the clot was removed by centrifuging at 1000× *g* for 10 min in a refrigerated centrifuge. Following centrifugation, the liquid supernatant (serum) was immediately transferred into a clean polypropylene tube using a Pasteur pipette. The samples were maintained at 2–8 °C while handling. If the serum did not analyzed immediately, the serum it was apportioned into 0.5 mL aliquots, and stored at –20 °C. For plasma preparation, whole blood was collected into commercially available anticoagulant EDTA-treated tubes. Cells were removed from plasma by centrifugation for 10 min at 1000× *g* using a refrigerated centrifuge. Following centrifugation, the liquid supernatant (plasma) was immediately transferred into a clean polypropylene tube using a Pasteur pipette. The samples should be maintained at 2–8 °C while handling. If the plasma was not analyzed immediately, the plasma was apportioned into 0.5 mL aliquots and stored at –20 °C. The liver and kidney samples were collected for biochemical assessments.

### 2.6. Measurement of Lipid Peroxidation in Serum

Measuring serum thiobarbituric acid reactive substances (TBARS), a quantitative measurement of lipid peroxidation, was quantified by their reaction with thiobarbituric acid (TBA) according to the method previously described [14]. In brief, samples (100 μL) were mixed with the equal volume of TBA reagent (0.67%, *w*/*w*) and placed in a boiling water bath for 30 min, and then centrifuged at 1811× *g* for 5 min. Collected the supernatants and measured its absorbance at 532 nm with an enzyme-linked immunosorbent assay (ELISA) plate reader (Quant, BioTek, Winooski, VT, USA).

### 2.7. Measurement of GSH and Total Thiol Levels in Plasma

A commercially available test kit from Randox Laboratories Ltd. (Antrim, UK) was used to evaluate the levels of glutathione (GSH) and total thiols in the plasma of experimental animals. The homogenate (720 μL) was double diluted, and 5% TCA was added to it to precipitate the protein content of the homogenate. After centrifugation (10,000× *g* for 5 min) at 4 °C the supernatant was taken, 5,5′-dithiolbis-2-nitrobenzoic acid (DTNB) solution was added to it and the absorbance was measured at 412 nm on a spectrophotometer. A standard graph was drawn using different concentrations of standard GSH solution (1 mg/mL). With the help of the standard graph, GSH contents in the homogenates of the experimental animals were calculated.

### 2.8. Measurement of Protein Carbonyls in Serum, Liver and Kidney

The quantification of the carbonyl protein assay, based on the reaction with dinitrophenylhydrazine, could be used to reflect the oxidative damage of the protein, as described previously [15]. Briefly, the samples (100 μL) were added with 20% trichloroacetic acid (30 μL) to precipitate the protein and redissolved in 10 mM dinitrophenylhydrazine (100 μL) to obtain a final protein concentration of 1–2 mg/mL, with 2 M HCl (100 μL) added to the corresponding sample aliquot reagent blanks. The absorbance was measured at 370 nm with an ELISA plate reader.

### 2.9. Measurement of SOD, Catalase, G6PD, GSH-Px and GSH-Rd Activities in Liver and Kidney

Organ homogenates (tissue: buffer ratio of 1:5, wet weight: volume of buffer) were prepared in a cold Tris buffer [5 mmol/L, containing 2 mmol/L ethylenediaminetetraacetic acid (EDTA), pH 7.4], utilizing a homogeniser with a 1500 rotatory speed of piston/min. The homogenates were centrifuged at 10,000× *g* for 10 min at 4 °C and the supernatants were collected and then used immediately to determine the activities of SOD, catalase, G6PD, GSH-Px and GSH-Rd, as per the manufacturer’s instructions on the Randox Laboratories Ltd. kit. For the SOD assay, reaction mixture contained 1.2 mL of sodium pyrophosphate buffer (0.052 mmol/L, pH 7.0), 0.1 mL of phenazine methosulfate (PMS) (186 μmol/L), 0.3 mL of nitro blue tetrazolium (NBT) (300 μmol/L). 0.2 mL of the supernatant obtained after centrifugation (1500× *g*, 10 min followed by 10,000× *g*, 15 min) of 10% kidney homogenate was added to reaction mixture. Enzyme reaction was initiated by adding 0.2 mL of NADH (780 μmol/L) and stopped precisely after 1 min by adding 1 mL of glacial acetic acid. Amount of chromogen formed was measured by recording color intensity at 560 nm. Results are expressed as units/mg protein. For the catalase assay, 50 μL potassium phosphate buffer (250 mmol/L, pH 7.0) was incubated with 50 μL methanol and 10 μL hydrogen peroxide (0.27%). The reaction was initiated by addition of 100 μL of enzyme sample with continuous shaking at room temperature (20 °C). After 20 min, reaction was terminated by addition of 50 μL of 7.8 mol/L potassium hydroxide. 100 μL of purpald (4-Amino-3-hydrazino-5-mercapto-1,2,4-triazole, 34.2 mmol/L in 480 mmol/L HCl) was immediately added, and the mixture was again incubated for 10 min at 20 °C with continuous shaking. Potassium peroxidate (50 μL 65.2 mmol/L) was added to obtain a colored compound. The absorbance was read at 550 nm in a spectrophotometer. For the GSH-Px assay, 100 μL of enzyme sample was incubated for 5 min with 1.55 mL stock solution (prepared in 50 mmol/L Tris buffer, pH 7.6 with 0.1 mmol/L EDTA) containing 0.25 mmol/L GSH, 0.12 mmol/L NADPH, and 1 unit glutathione reductase. The reaction was initiated by adding 50 μL of cumene hydroperoxide (1 mg/mL), and the rate of disappearance of NADPH with time was determined by monitoring absorbance at 340 nm. One unit of enzyme activity is defined as the amount of enzyme that transforms 1 μmol of NADPH to NADP per min.

### 2.10. Statistical Analysis

All data are expressed as means ± standard deviation (SD), and the significance of the differences between groups was performed using one-way analysis of variance (ANOVA), followed by Dunnett multiple comparison test. A *p* value < 0.05 was considered as statistical significance.

## 3. Results

### 3.1. Catechin Content in Green Tea Extract

**Figure 1:**
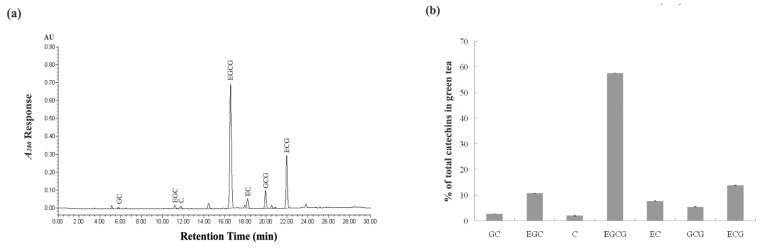
Major components of green tea extract in this study. (**a**) HPLC chromatogram of catechins from green tea extracts. (**b**) Major components of green tea extract were determined as a percentage of the total catechins. Each value is expressed as mean ± SD of three replicates. GC, (+) -gallocatechin; EGC, (−) -epigallocatechin; C, (+) -catechin; EGCG, (−) -epigallocatechin gallate; EC, (−) -epicatechin; GCG, (−) -gallocatechin gallate; ECG, (−) -epicatechin gallate.

The catechins present in green tea extract from HPLC analysis are presented in Figure 1. The seven catechins in the green tea extract analyzed in this study could be completely separated within 30 min (Figure 1a), and the total content of the catechins in the green tea extract was 258.55 ± 4.51 mg/g green tea extract. The most abundant catechin in green tea extract was EGCG (150.37 ± 2.41 mg/g green tea extract), which accounts for 58.16% of the total catechin (the sum of this study-mentioned seven catechins), followed by ECG and EGC (35.37 ± 0.13 mg/g green tea extract and 26.73 ± 0.97 mg/g green tea extract, respectively), which account for 13.68% and 10.34% of the total catechin, respectively (Figure 1b). Other important catechins in green tea extract were GC, C, EC and GCG.

### 3.2. Effects of Green Tea Extract on Lipid Peroxidation, Protein Carbonyls, Total Thiols and GSH in Blood

The effects of green tea extract on the levels of lipid peroxidation and protein carbonyls in the serum and the levels of total thiols and GSH in the plasma are summarized in Table 1. The aged control group had significantly higher serum levels of TBARS and protein carbonyls and lower plasma levels of total thiols and GSH than did in the young control group (*p* < 0.05), suggesting that senescence regulated an increase of serum lipid peroxidation and protein oxidative damage and declined the nonenzymatic antioxidants in the plasma. However, treatment with green tea extract at doses of 125, 625 and 1250 mg/kg significantly diminished serum levels of TBARS and protein carbonyls compared with the aged control group. Moreover, there were significant increases (*p* < 0.05) in the levels of plasma total thiols and GSH in the green tea extract-treated group at all the test doses compared to the aged control group. The results of the blood biochemical analysis showed that green tea extract might provide protection against blood redox imbalance during aging, not only reduced lipid peroxidation and protein oxidative damage, but also increased or maintained intracellular antioxidants.

### 3.3. Green Tea Extract Supplementation Ameliorates the Senescence-Mediated Protein Oxidative Damage in the Liver and Kidney

**Figure 2:**
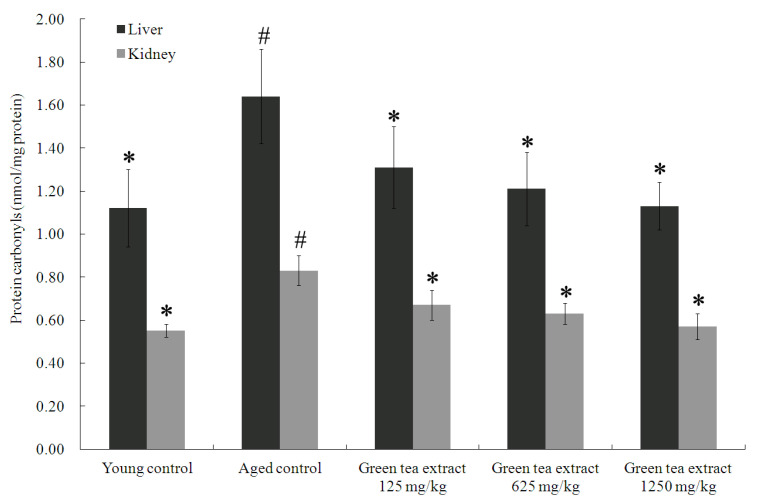
Effect of green tea extract on protein carbonyls in the liver and kidney of aged mice. Values are the mean ± SD for ten mice; # *p* < 0.05 compared with the young control; * *p* < 0.05 compared with the aged control.

In order to evaluate the effect of green tea extract treatment on aging-induced protein oxidative damage in the organs, this study analyzed the protein carbonyl levels in the liver and kidneys, as shown in Figure 2. The livers and kidneys of the aged control group displayed significantly elevated levels of protein carbonyl levels (1.64 ± 0.22 nmol/mg protein and 0.83 ± 0.07 nmol/mg protein, respectively), compared to the young control group (1.12 ± 0.18 nmol/mg protein and 0.55 ± 0.03 nmol/mg protein, respectively) (*p* < 0.05). On the contrary, green tea extract supplementation markedly attenuated senescence-mediated protein oxidative damage in the liver and kidney. Green tea extract supplementation at doses of 125, 625 and 1250 mg/kg significantly diminished the percentage of protein carbonyls in the liver (1.31 ± 0.19 nmol/mg protein, 1.21 ± 0.17 nmol/mg protein, and 1.13 ± 0.11 nmol/mg protein, respectively), compared to the aged control group (*p* < 0.05). Similar results were also found in the kidney (Figure 2). Treatment with green tea extract at doses of 125, 625 and 1250 mg/kg significantly diminished the percentage of protein carbonyls in the (0.67 ± 0.07 nmol/mg protein, 0.63 ± 0.05 nmol/mg protein, and 0.57 ± 0.06 nmol/mg protein, respectively), compared to the aged control group (*p* < 0.05).

### 3.4. Green Tea Extract Supplementation Ameliorates the Senescence-Mediated Hepatic Antioxidant Enzyme Activities

**Figure 3:**
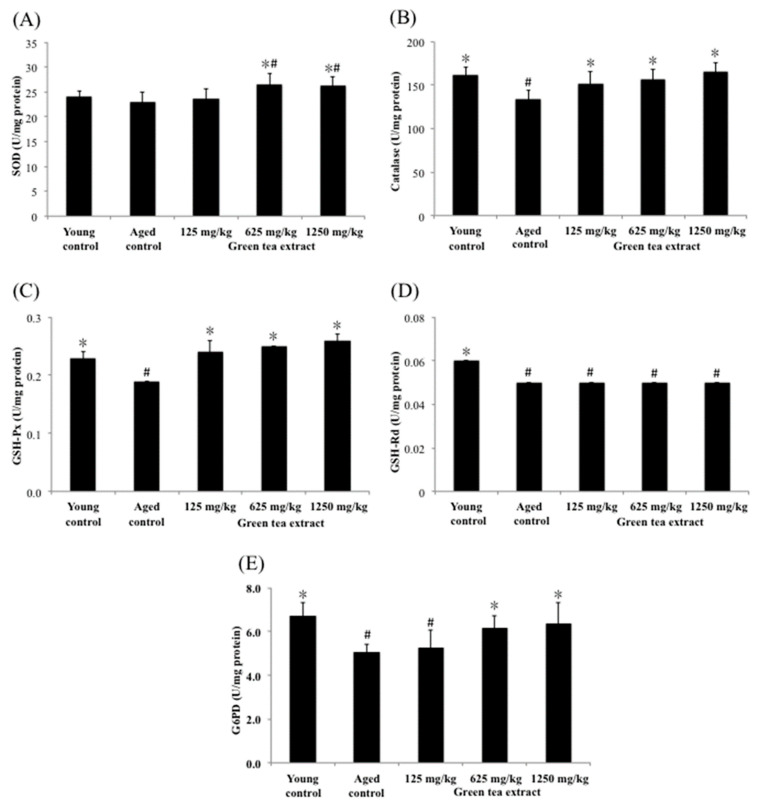
Effect of green tea extract on liver SOD, catalase, GSH-Px, GSH-Rd and G6PD of aged mice. (**A**) SOD activity. (**B**) catalase activity. (**C**) GSH-Px activity. (**D**) GSH-Rd activity. (**E**) G6PD activity. Values are the mean ± SD for ten mice; # *p* < 0.05 compared with the young control; * *p* < 0.05 compared with the aged control.

The tissues or organs of aging organisms are susceptible to increased oxidative stress and affect their functions. Part of the reason is that their ability to remove reactive oxygen molecules produced by metabolism is much lower than that of younger populations. However, the antioxidant defense function in cells declines during aging, which can be strengthened by supplementing antioxidants in the daily diet. In this study, the effects of green tea extract on the activities of hepatic SOD, catalase, G6PD, GSH-Px and GSH-Rd were measured as indicators of intracellular antioxidant status and the results are shown in Figure 3. The levels of hepatic catalase, G6PD, GSH-Px and GSH-Rd in the aged control group (133.42 ± 10.49 U/mg protein, 5.03 ± 0.40 U/mg protein, 0.19 ± 0.00 U/mg protein, and 0.05 ± 0.00 U/mg protein, respectively) were significantly lower than those in the young control group (162.25 ± 8.85 U/mg protein, 6.72 ± 0.60 U/mg protein, 0.23 ± 0.01 U/mg protein, and 0.06 ± 0.00 U/mg protein, respectively) (*p* < 0.05), suggesting senescence-mediated diminutions in antioxidant enzymes activities in the liver. In contrast to the aged control group, mice treated with minimum dose of green tea extract (125 mg/kg) significantly increased (*p* < 0.05) the activities of catalase and GSH-Px, but did not significantly affect SOD, G6PD and GSH-Rd compared to the aged control group. Fortunately, the group that was treated with the maximum dose of green tea (1250 mg/kg) showed significantly increased activity of SOD, catalase, G6PD and GSH-Px (26.24 ± 1.85 U/mg protein, 165.93 ± 10.22 U/mg protein, 6.38 ± 0.95 U/mg protein, and 0.22 ± 0.01 U/mg protein, respectively) in the liver of aged mice (*p* < 0.05). A similar result was found in the group that was treated with the dose of 625 mg/kg of green tea extract.

### 3.5. Green Tea Extract Supplementation Ameliorates the Senescence-Mediated Kidney Antioxidant Enzyme Activities

**Figure 4:**
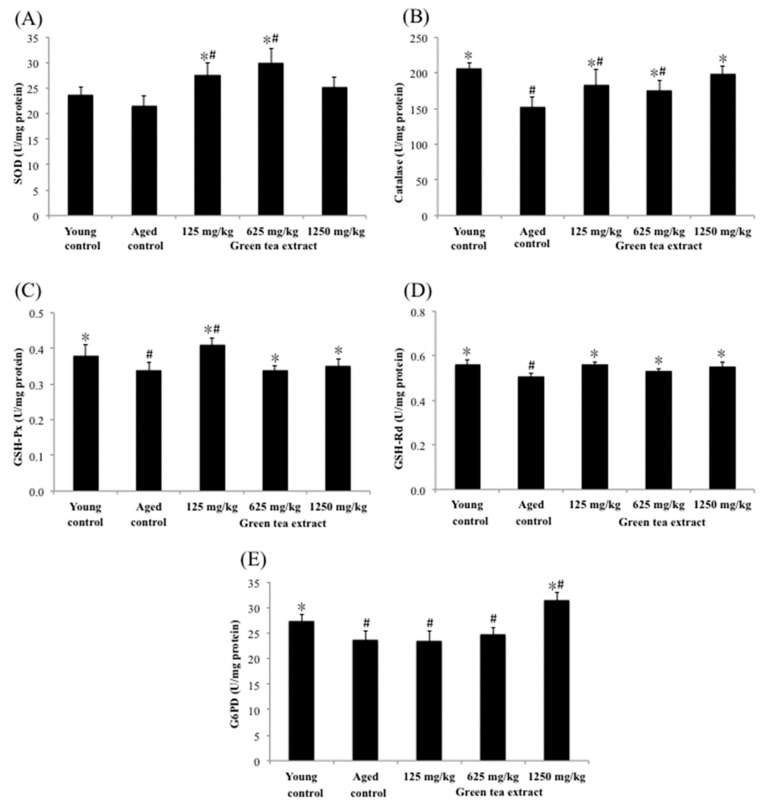
Effect of green tea extract on kidney SOD, catalase, GSH-Px, GSH-Rd and G6PD of aged mice. (**A**) SOD activity. (**B**) catalase activity. (**C**) GSH-Px activity. (**D**) GSH-Rd activity. (**E**) G6PD activity. Values are the mean ± SD for ten mice; # *p* < 0.05 compared with the young control; * *p* < 0.05 compared with the aged control.

Figure 4 shows the effects of green tea extract on the activities of renal antioxidant enzymes. The activities of renal catalase, G6PD, GSH-Px and GSH-Rd in the aged control group were significantly diminished (152.28 ± 13.84 U/mg protein, 23.69 ± 1.72 U/mg protein, 0.34 ± 0.02 U/mg protein, and 0.51 ± 0.01 U/mg protein, respectively) (*p* < 0.05), compared with the young control group (206.43 ± 8.51 U/mg protein, 27.50 ± 1.24 U/mg protein, 0.38 ± 0.03 U/mg protein, and 0.56 ± 0.02 U/mg protein, respectively). However, mice treated with 125 mg/kg of green tea extract significantly increased (*p* < 0.05) the renal activity of SOD, catalase, GSH-Px and GSH-Rd (27.51 ± 2.39 U/mg protein, 183.24 ± 21.11 U/mg protein, 0.41 ± 0.02 U/mg protein, and 0.56 ± 0.01 U/mg protein, respectively), compared to the aged control group. Similar results were also found in the dose of 625 mg/kg of green tea extract. In particular, the maximum dose of green tea extract (1250 mg/kg) administered to mice not only increased the aforementioned antioxidant enzymes, but also significantly increased the activity of G6PD in the kidney by 33%.

## 4. Discussion

Many animal experiments have confirmed that the antioxidant status of various organs in aging mice was significantly lower than that of young mice, including the decrease in the function of the antioxidant defense system and the increase in the level of lipid peroxidation. Therefore, it is necessary to intake the natural antioxidants from the daily diet to enhance the ability of the antioxidant defense system in vivo, or to assist the antioxidant defense system to fight oxidative damage mediated by aging. Green tea polyphenols are natural antioxidants that have health-promoting effects. The biological activities of green tea have been reported in many in vitro and in vivo studies. Most studies have shown that the beneficial effects of green tea are due to its rich polyphenols. Among them, the most abundant EGCG has the most excellent antioxidant capacity [7].

However, some reports about the adverse effects of green tea polyphenols have shown that the ingestion of large amounts of green tea polyphenols, especially when the intestinal mucosa is damaged, is a matter of controversy [16,17]. Another study also pointed out that the co-administration of the selenite, an essential trace element, and EGCG resulted in the mortality of treated mice in a dose and time-dependent manner [18]. Additionally, in vitro studies reported that administration of rat hepatocytes with high concentrations of EGCG resulted in reduced cell viability [19,20]. In vivo studies also suggest that administration with a single dose of 1500 mg/kg, of EGCG in mice may result in hepatotoxicity [21]. Due to numerous interactions and synergisms, it is difficult to study the effects of natural dietary supplements on human health when administered in complex mixtures as opposed to a purified compound. In the present study, the seven catechins in the green tea extract analyzed in this study could be completely separated within 30 min. Our results were consistent with several previous studies [12,13] showing that green tea leaf extracts were rich in catechins, and the main components of catechins in green tea extract were EGCG, EGC and ECG, among which EGCG was containing the highest proportion, accounting for more than half of the total catechins. Additionally, previous studies have shown that the recommended intake of green tea that have beneficial to human health is at least three cups a day, providing at least 250 mg/day of catechins [22,23]. Therefore, the doses used in this study (125, 625, and 1250 mg/kg/day) were equivalent to 0.5–5 times the recommended daily intake.

TBARS is a mixture of lipid hydroperoxides and aldehydes in various experimental animals’ tissue and serum, usually expressed as the equivalent of malondialdehyde (MDA), which is the major reactive aldehyde produced during the peroxidation of biological membrane polyunsaturated fatty acids with increasing of age and oxidative stress [24,25]. Moreover, practically all types of ROS, including free radical species and non-radical species, can induce the oxidative cleavage of proteins by glutamyl side chain oxidation or α-amidation pathways, leading to the generation of carbonyl groups on protein side chains. As we all know, the oxidation of lipids and proteins produces lipid peroxides and protein carbonyl groups, respectively, leading to the initiation of various tissue damages and aging related to oxidative stress, as well as involving the pathogenesis of many age-related degenerative diseases [24,26]. In the present study, we have found an increase on the serum levels of TBARS and protein carbonyl in aged mice. The most abundant catechins contained in green tea extract scavenge a wide range of ROS, which may initiate lipid and protein oxidative modifications. Therefore, green tea extract may decrease the formation of ROS and terminate the initiation and expansion of lipid peroxidation and protein oxidation. Our results are in agreement with previous studies that administration of green tea extract to aged rats resulted in significantly reduction of TBARS and protein carbonyl levels [11].

GSH and thiol groups are very effective intracellular non-enzymatic antioxidants, which can reduce the ROS and xenobiotics harmfulness. The depletion of intracellular GSH is mainly due to the conjugation with xenobiotics or ROS to be oxidized to glutathione disulfide (GSSG). Subsequently, GSSG either rapidly reduces by the catalytic reaction of GSH-Rd and NADPH to form GSH, or it is used in the endoplasmic reticulum to help the protein folding process. Hence, GSH is a particularly effective intracellular antioxidant for detoxifying or scavenging ROS against oxidative stress [27]. Total thiols are composed of three forms in organisms, including two disulphides and free thiols, and its function has been confirmed not only as a strong antioxidant that can defend against the damage of free radicals, but also as an important player at the molecular level, such as participating in detoxification, regulating signal transduction, mediating cell apoptosis and various other functions. Although the state of glutathione and total thiols in vivo declined due to aging, they are of great significance in protecting cells or organs from the threat of oxidative stress in the aging process. An in vivo study indicated that there were no significant changes observed on cellular redox status in young rats with green tea extract supplementation. However, green tea extract administration was found to be very effective in reducing lipid peroxidation and protein carbonyl and improving enzymatic and non-enzymatic antioxidants and redox status in aged rats [28]. The outcome of this study found that the plasma levels of GSH and total thiols had significantly diminished in the aged mice than did in the young mice, suggesting that senescence regulated a diminution of nonenzymatic antioxidants. Fortunately, significantly higher plasma levels of GSH and total thiols were found in the green tea extract groups than the aged mice group, which is in agreement with previous reports by Assunção et al. [27]. Their study indicated that the green tea treatment group had significantly higher GSH and lower GSSG levels compared with the age-matched group.

ROS generated under normal aerobic metabolism can cause protein oxidative damage through glutamyl side chain oxidation, and then form protein carbonyls that are the most common biomarker of protein oxidation [15]. An in vivo study indicated that the hepatic and renal protein carbonyls contents were significantly elevated in the aged control rats compared to the young control rats [29]. The result of this study is consistent with our current research, showing that senescence-mediated protein oxidative damage in organs. Otherwise, the administration of green tea extract significantly diminished the protein carbonyls levels in the liver and kidney relative to the aged control group. Similar results from previous research confirmed that the administration of green tea can protect cells and tissues from oxidative damage by scavenging oxygen free radicals and can significantly diminish the hepatic levels of protein carbonyls groups caused by ethanol in the aged rats [11].

To prevent the oxidative damage caused by the increase in oxidative stress, the organism has built an antioxidant defense system, which includes antioxidants (such as glutathione, bilirubin, vitamin C and α-tocopherol) that can remove harmful active metabolites and detoxifying antioxidant enzymes (such as SOD, catalase, G6PD, GSH-Px and GSH-Rd) in protecting cells and organs from all kinds of oxidative damage induced by free radicals during aging [30]. In terms of preventing aging-mediated redox imbalance, maintaining or enhancing the activity of enzymatic antioxidants and/or inhibiting the generation of free radicals are very important strategies [30]. However, the increase in lipid peroxides or free radicals can easily reduce the activities of these enzymatic antioxidants, leading to the breakdown of these enzymatic antioxidants, and thus the decline of organ functions. Studies have pointed out that the increase in the incidence of liver disease in the elderly people is age-related, associated either with the increase in free radicals or the decrease in liver antioxidant enzymes activities [25]. Animal studies revealed that liver antioxidant enzymes, including SOD, catalase, GSH-Px, and GSH-Rd, were mediated by aging, leading to reduced activities, which in turn impairs liver function [25,31]. Rikans et al. [32] reported that the antioxidant enzyme activities (catalase and GSH-Rd) in post-mitochondrial supernatants prepared from livers were significantly diminished in aged male and female Fischer 344 rats, but SOD and GSH-Px activities were unaffected by aging. Our study also clearly displayed that oral administration of green tea extract to aged mice for 4 weeks significantly increased the hepatic levels of SOD, catalase, G6PD and GSH-Px, that was diminished by aging in mice. Our results together with several previous findings suggested that dietary supplementation with green tea extract could be protective against the senescence-related inactivation of hepatic antioxidant enzymes [10,33,34]. Furthermore, one of the mechanisms about the ability of green tea to upgrade the activity of hepatic enzymes may be that it can effectively remove ROS or xenobiotics and inhibit the production of lipid peroxides [35].

Most clinical studies on the involvement of the kidneys in the aging process have focused on the study of the effects of aging on chronic kidney disease. A clinical study in China reported that the increase in prevalence of chronic kidney disease with increasing age was observed in both males and females of the elderly population of Beijing [36]. In vivo, in rodents, the kidneys in the aged animals are subjected to a harsher oxidative environment than the young, which damages the antioxidant status of the kidneys and causes redox imbalance. They also suggested that the activities of SOD and GSH-Px in the kidneys were significantly diminished during aging [37]. Fortunately, the decline in antioxidant defenses against oxidative stress during aging can be enhanced or maintained by supplementing extracellular antioxidants [26]. In addition, the green tea tannins could also help the residual kidneys to scavenge free radicals in rats with nephrectomy [28]. The results in this study, consistent with previous in vivo studies, have shown that the antioxidant status in the kidneys of aging mice would be significantly deteriorated during the aging process, such as the antioxidant enzymes activities of the kidneys that were significantly reduced. However, administration of green tea extract significantly restored the antioxidant enzymes activities in the kidney. These results have similar conclusions to previous studies, which confirmed that green tea extract has a positive effect on reducing oxidative stress and restoring or increasing the activities of enzymes such as SOD, catalase and G6PD in the kidneys [33].

Although the amount of catechins present in green tea extract is well documented, the question of the bioavailability and metabolism of green tea catechins in vivo remains uncertain, as the polyphenol’s absorption, metabolism, bioavailability, tissue distribution, intracellular accumulation and excretion are more complicated in vivo. Several studies have been reported stating that green tea polyphenols have various metabolites, based on evidence that intestinal microorganisms metabolize polyphenols through ring fission, hydrolysis, methylation, oxidation and conjugation with glucuronide and sulphate [38,39]. Only a few papers had reported on the metabolism of green tea catechins in animal studies and in human subjects after administration of green tea catechins. For instance, sulphate forms and glucuronide forms of catechins (EGCG, EGC and EC) were found in plasma and urine after oral administration of green tea extract to healthy volunteers [40]. Another study has been reported that another conjugate form of EGCG, EGCG 4′-mono-sulphate, is metabolized by arylsulfotransferase of human intestinal bacteria [41]. A LC-MS study indicated that five (-)-epicatechin metabolites could be measured in human plasma after 1 h of administration and the major epicatechin metabolites were the glucuronides of epicatechin and its 3′-O-methylated metabolite, 3′-O-methylated epicatechin glucuronide [42]. However, the glucuronides may be cleaved in vivo by β-glucuronidases, which are present in a number of tissues within the body [43]. Additionally, accurate quantification of targets at extremely low levels in the biomatrix has frequently relied on the use of stable isotope-labeled (or radiolabeled) compounds for reliable assessment of the whole-body distribution in organism. Kohri et al. (2001) found that oral administration of [4-^3^H] EGCG to rats is thus absorbed by and has found wide-ranging distribution of radioactivity in their organs, including the liver and kidneys [44]. Another study of green tea catechins distribution in mice indicated that duplicate administration [^3^H]-EGCG enhances the level of radioactivity in blood, liver, kidney, brain and several other tissues, at 4–6 times above those after a single administration [39], suggesting that repeated feeding of green tea catechins enables the body to maintain a high-level of green tea catechins. Collectively, these results indicate that it is difficult to accurate by quantify individual catechins in the brain of mice, due to extremely complication of metabolism and biotransformation in vivo that have not been fully understood. However, it is sure that green tea catechins localise within the liver and kidney tissue, suggesting that they are candidates for direct protective action in the liver and kidney.

## 5. Conclusions

Although some articles have pointed out that the effect of the administration of large amounts of green tea polyphenols is a controversial matter, it is undeniable that taking a moderate amount of green tea polyphenols has a good effect on the human body. In particular, compared with other natural antioxidants, green tea polyphenols have an advantage of being easily obtained as a water-soluble natural antioxidant. Many studies have pointed out that green tea extract has the function of enhancing the antioxidant defense system and reducing lipid peroxidation in various organs of many aging animals [2,15]. This study demonstrated that green tea extract had significant antioxidant activity due to its rich polyphenols and had a significant inhibitory effect on aging-mediated oxidative damage in aged mice, as evidence of not only diminishing TBARS and protein carbonyl levels in the serum, but also of increasing levels of GSH and total thiols in the plasma. In the meantime, administration of green tea extract significantly inhibited the levels of protein carbonyls and increased the intracellular antioxidant enzymes activities in the livers and kidneys of aged mice. Overall, green tea is an easy-to-drink and very effective source of water-soluble natural antioxidants, which can regulate the intracellular antioxidant balance and furnish predictable health benefits for the liver and kidneys during the aging process.

## Figures and Tables

**Table 1:** Effect of green tea extract on lipid peroxidation, protein carbonyls, total thiols and GSH in blood of aged mice.

Design of Treatment	Young Control	Aged Control	Green Tea Extract		
			125 mg/kg	625 mg/kg	1250 mg/kg
TBARS (nmol/μg protein) ^1^	70.1 ± 3.24 *	74.3 ± 4.71 ^#^	69.6 ± 3.90 *	66.3 ± 4.15 *	61.9 ± 3.23 ^#^ *
Protein carbonyls (nmol/mg protein) ^1^	0.79 ± 0.04 *	1.33 ± 0.10 ^#^	1.09 ± 0.11 ^#^ *	0.80 ± 0.07 *	0.96 ± 0.06 ^#^ *
Total thiols (μmole/mg protein) ^2^	3.43 ± 0.14 *	2.75 ± 0.18 ^#^	2.56 ± 0.28 ^#^	3.07 ± 0.25 ^#^ *	3.34 ± 0.25 *
GSH (μmole/mg protein) ^2^	0.30 ± 0.02 *	0.23 ± 0.01 ^#^	0.26 ± 0.01 ^#^ *	0.25 ± 0.01 ^#^ *	0.26 ± 0.02 ^#^ *

Values are mean ± S.D., n = 10/group. ^1^ The tests were determined in serum of experimental animals. ^2^ The tests were determined in plasma of experimental animals. ^#^ *p* < 0.05 compared with young control. * *p* < 0.05 compared with aged control.

## Data Availability

The data presented in this study are available on request from the corresponding author.
